# The occurrence of cervical high-grade lesions in women outside the recommended age screening

**DOI:** 10.61622/rbgo/2025rbgo41

**Published:** 2025-09-08

**Authors:** Thatiana Terzi Galvão Pavarino, Isabel Cristina Chulvis do Val Guimarães, Caroline Alves Oliveira, Susana Cristina Aidé Viviani, Fabiana Resende Rodrigues

**Affiliations:** 1 Universidade Federal Fluminense Faculdade de Medicina Departamento de Ginecologia e Obstetrícia Niterói RJ Brazil Departamento de Ginecologia e Obstetrícia, Faculdade de Medicina, Universidade Federal Fluminense, Niterói, RJ, Brazil.

**Keywords:** Human papillomavirus, Uterine cervical dysplasia, Squamous intraepithelial lesions, Persistent infection

## Abstract

**Objective:**

This study aimed to assess the prevalence of cervical High-Grade Squamous Intraepithelial Lesions (HSIL) in women outside the screening age recommended by the Brazilian Guidelines for Cervical Cancer Screening (under 25 and over 64 years old).

**Methods:**

The cross-sectional study was conducted at a reference hospital in Rio de Janeiro with a histopathological report of CIN 2 + from January 2010 to December 2020 through the analysis of medical records.

**Results:**

Among 406 women diagnosed with Cervical Intraepithelial Neoplasia (CIN) 2+, 63 patients (15.5%) were outside the recommended screening age, 17(4.2%) of whom were under the age of 25, and 46 (11.3%) were older than 64 years. CIN 2 was most prevalent in women under 25 years old (29.4%); CIN 3 in those between 25 and 64 years old (55.1%); and invasive cancer predominated in women over 64 years old, with statistical significance (<0.001).

**Conclusion:**

The higher frequency of CIN 2 in young women under 25 years old supports the transient nature of these lesions, reinforcing that screening this age group may lead to unnecessary treatment. Conversely, the detection of high-grade lesions and cancer in older women is a consequence of inadequate screening earlier in life.

## Introduction

Cervical cancer is one of the most prevalent malignancies in women worldwide and remains a major public health concern, particularly in developing countries. In Brazil, with the exception of non-melanoma skin cancer, cervical cancer is the third most common cancer, and the fourth leading cause of death from cancer in women. The National Cancer Institute estimated that approximately 17,010 new cases would be diagnosed in 2023, and in 2020, 6,627 deaths were recorded.^(1)^ In Brazil, cervical cancer incidence is closely linked to papillomavirus **(**HPV) infection and inadequate screening coverage**.**

Decisions about how, who, and when to screen for cervical cancer and its precursor lesions in asymptomatic populations require a thorough analysis of the benefits, harm, and costs. In Brazil, the predominant pattern of screening is opportunistic, i.e., women have undergone a Papanicolaou smear (Pap smear) when seeking health care for other reasons. As a result, 20% to 25% of the tests have been carried out outside the recommended age range and approximately half of these were performed at intervals of one year or less. This contrasts with Brazilian national guidelines, which advise screening between the ages 25 to 64 years, with cytology conducted every three years following two consecutive normal results.^(2)^ Consequently, there is a contingent of women who are over-screened and another contingent who are not screened at all.

Cytological screening should continue until age 64 and should be discontinued in women with no previous history of preinvasive neoplastic disease if they have had at least two consecutive negative tests in the last five years. For women over 64 years of age who have never had cytopathology, two tests should be performed one to three years apart before stopping screening.^(2)^

The main objective of this study was to evaluate the occurrence of cervical High-Grade Squamous Intraepithelial Lesions (HSIL) in women outside the age group recommended by the Brazilian guidelines for cervical cancer screening, i.e., under 25 and over 64 years of age. The secondary objectives were to evaluate and compare the demographic and epidemiological data (sexual debut, number of sexual partners, parity, consistently condom use, estrogen contraception, immunosuppression, smoking) among the age groups recommended and not recommended for cervical cancer screening; to evaluate and compare the source cytology with the histologies of CIN2+ in the age groups recommended and not recommended.

## Methods

This cross-sectional study included women from the Colposcopy Unit Genital Tract Pathology and Colposcopy Department, Gynecology Service of Antônio Pedro University Hospital (HUAP), and Fluminense Federal University (UFF) with a histopathological report of CIN2+ between 2010 and 2020 through the analysis of medical records. During this period, cervical cancer screening guidelines and diagnostic methods within Brazil's public healthcare system remained relatively stable.

Access was granted to study the medical records of women with a histopathologic diagnosis of HSIL or cancer after colposcopy-guided biopsy as well as a review of the pathologist's report. From a population of 4,155 women enrolled in the Gynecology Department at HUAP, 524 women (12.6%) were diagnosed with high-grade cervical squamous intraepithelial lesions or cervical cancer. Of these, 118 medical records (22.5% of the 524) were excluded from the study. Specifically, 75 records (14.3%) were inaccessible, with 68 (13%) untraceable due to a system transition from physical to electronic records at HUAP, 12 (2.3%) containing incorrect registration numbers, and 5 (1%) belonging to deceased patients whose records were unavailable. Additionally, 33 records (6.3%) lacked histological confirmation by the HUAP Pathology Department, as biopsies were performed in another center. After applying these exclusions, a total of 406 women (77.5% of the initial 524) were included in the study, among whom 17 (4.2%) were under 25 years of age and 46 (11.3%) were over 65 years.

The variables studied were: Histopathologic diagnosis of Cervical Intraepithelial Neoplasia (CIN) CIN 2, CIN 3, and cancer; age over 64 and under 25; age of sexual debut (this was considered early if it occurred before the age of 14); number of sexual partners; parity (women with three or more pregnancies were considered multiparous); consistently condom use; use of estrogen-containing contraceptives; presence of immunosuppression (women living with HIV, solid organ transplant patients, autoimmune disease patients without limitations); smoking; cytology result.

The IBM SPSS software version 20.0 was used for statistical analysis. Categorical data were described by absolute frequencies and percentages, and numerical data by median and quartiles, since they did not have a normal distribution, which was verified by the Shapiro-Wilk test. Categorical variables were compared among the three groups (CIN 2, CIN 3 and cancer) using Pearson's asymptotic chi-squared test (20% of the expected value less than 5 and 80% of the expected value greater than 5) and Pearson's exact chi-squared test (more than 20% of the expected value less than 5). If the Pearson chi-squared test showed statistical significance in tables larger than 2x2, standardized adjusted residuals were analyzed to identify the associations. Residuals ≥ +1.96 indicated a higher frequency in a category, while residuals ≤ -1.96 indicated a lower frequency. The Mann-Whitney test was used to compare numerical variables between groups because the data did not follow a normal distribution, as confirmed by the Shapiro-Wilk test. The Kruskal-Wallis test was used when there were more than two groups, and the Mann-Whitney test with Bonferroni correction was used when there was statistical significance. Statistical significance was set at 5%.

This study was conducted in accordance with all applicable guidelines and regulations on human subject research in Brazil as per National Health Council Resolution 466/2012. The project was approved by HUAP's Ethics Committee for Research Involving Human Beings 5.083.505 (*Certificado de Apresentação de Apreciação Ética:* 52478721.5.0000.5243).

## Results

A total of 406 women were included in this study. The median age was 44 years old. The median age of sexual intercourse was 17 years old, and the median number of sexual partners was three. Among the 406 patients included in this study, diagnosed with CIN 2+, 4.2% (17/406) were younger than 25 years old, 84.5% (343/406) were between 25 and 64 years old, and 11.3% (46/406) were older than 64 years. In fifty-four (14.8%) women, sexual debut was before the age of 14. Twenty-six (7.5%) women had more than ten sexual partners during their lifetime. Immunosuppression was identified in 8.9% of patients in the study (Table 1).

**Table 1:** Characteristics of the categorical variables of women with a histological diagnosis of CIN2+

Categorical variables		n(%)
Sexual debut		
	Under or equal to 14 years old	54(14.8)
	Over 14 years old	310(85.2)
Number of sexual partners		
	More than 10	26(7.5)
	Less than or equal to 10	320(92.5)
Multiparity		
	Yes	228(58.5)
	No	162(41.5)
Condom use		
	Yes	36(9.7)
	No	335(90.3)
Use of estrogen-containing contraceptives		
	Yes	73(19.3)
	No	306(75.4)
Immunosuppression		
	Yes	35(8.9)
	No	357(91.1)
Smoking		
	Yes	151(38.8)
	No	238(61.2)

We evaluated the cytology results of the 406 women according to histologic grade (CIN 2, CIN 3 and cancer). Of the 46 women with a histopathologic diagnosis of CIN 2, only one (2.2%) had negative source cytology for neoplasia, 3(6.5%) had Atypical squamous cells of undetermined significance, possibly non-neoplastic (ACS-US), 4(8.7%) Atypical squamous cells of undetermined significance in which high-grade lesion cannot be excluded (ASC-H), 8(17.4%) Low-grade squamous intraepithelial lesion (LSIL), 29(63%) HSIL, and 1(2.2%) invasive adenocarcinoma (Table 2). Among the 210 women (51.7%) with CIN 3, source cytology showed 1 (0.5%) negative for neoplasia, 8 (3.8%) ASC-US, 12 (5.7%) ASC-H, 7 (3.3%) LSIL, 166 (79%) HSIL, 2 (1%) AGC, 10 (4.8%) epidermoid carcinoma, 1 (0.5%) invasive adenocarcinoma, and 3 unknown cases (Table 2). Among the 150 women (36.9%) with a histopathologic diagnosis of cancer, 4(2.7%) had negative cytology for neoplasia, 1(0.7%) had ASC-US, 6(4.0%) ASC-H, 19(12.7%) HSIL, 1(0.7%) AGC, 45(30.0%) epidermoid carcinoma, while 1 (0.7%) presented with ASC-US, 7(4.7%) invasive adenocarcinoma, and for 66(44.0%) women the cytology results was unknown (Table 2). HSIL was the most frequent precursor lesion across all histologic grades (63% in CIN 2, 79% in CIN 3, 12.7% in cancer).

**Table 2:** Source cytology in cases of CIN2, CIN3 and cancer histopathology in a total of 406 women

Source cytology	CIN2 n=46 n(%)	CIN3 n=210 n(%)	Cancer histopathology n=150 n(%)
Negative	1(2.2)	1(0.5)	4(2.7)
ASC-US	3(6.5)	8(3.8)	1(0.7)
ASC-H	4(8.7)	12(5.7)	6(4.0)
LSIL	8(17.4)	7(3.3)	0(0.0)
HSIL	29(63.0)	166(79.0)	19(12.7)
AGC	0(0.0)	2(1.0)	1(0.7)
Epidermoid carcinoma	0(0.0)	10(4.8)	45(30.0)
Adenosquamous carcinoma	0(0.0)	0(0.0)	1(0.7)
Adenocarcinoma	1(2.2)	1(0.5)	7(4.7)
Unknown	0(0.0)	3(1.4)	66(44.0)

Atypical squamous cells of undetermined significance, possibly non-neoplastic (ASC-US); Atypical squamous cells of undetermined significance in which high-grade lesion cannot be excluded (ASC-H); Low-grade squamous intraepithelial lesion (LSIL); High-grade squamous intraepithelial lesion (HSIL); Atypical glandular cells of undetermined significance (AGC)

An analysis of the numerical and categorical variables was carried out comparing the age group recommended with that not recommended by the Brazilian Guidelines for Cervical Cancer Screening (Table 3). In the two-by-two comparison for the variable number of pregnancies: a higher ratio was observed in women aged 25 to 64 when compared to the group aged under 25. When comparing the group aged 25 to 64 with the group aged over 64, a higher ratio of the variable was observed with the older group. Both were statistically significant. In the two-by-two comparison for the sexual debut variable, there was a difference in the median between the groups aged over 64 and between 25 and 64, with the median being higher for the older group, with statistical significance. There was a difference between the proportions for the multiparity variable, with the proportion of multiparous women being higher in the group aged over 64, with statistical significance. There was a higher proportion of condom use in the group of women younger than 25 years, as well as for the variable use of estrogen contraceptives, with statistical significance. There was a higher proportion of smoking in the group aged between 25 and 64, with statistical significance. For the other variables, there was no difference at the 0.05 level.

**Table 3:** Comparison of demographic variables between women aged < 25, those between 25 and 64 years old, and patients > 64 years old

Variables		CIN2+ < 25 y n=17 n(%)	CIN2+ 25 to 64 y n=343 n(%)	CIN2+ > 64 y n=46 n(%)	p-value
No. of pregnancies (median (Q1; Q3)		1.0(0.0; 2.0)	3.0(2.0; 4.0)	5.0(3.0; 8.3)	0.004
Sexual debut (median (Q1; Q3)		16.0(15.0; 17.0)	17.0(15.0; 18.0)	18.0(16.0; 21.0)	<0.001
Sexual debut					
	>14 years old	14(87.5)	260(83.6)	36(97.3)	0.083*
	<14 years old	2(12.5)	51(16.4)	1(2.7)	
No. of partners					
	>10	1(5.9)	24(7.0)	1(2.2)	0.467*
	<10	16(94.1)	319(93.0)	45(97.8)	
Multiparity					
	Yes	2(11.8)*	189(55.1)	37(80.4)**	<0.001*
	No	15(88.2)**	154(44.9)	9(19.6)*	
Condoms					
	Yes	6(35.3)**	30(8.7)	0(10.0)*	<0.001*
	No	11(64.7)*	313(91.3)	46(100.0)**	
Estrogen contraception					
	Yes	8(47.1)**	64(18.7)	1(2.2)*	<0.001*
	No	9(52.9)*	279(81.3)	45(97.8)**	
Immunosuppression					
	Yes	1(5.9)	33(9.6)	1(2.2)	0.200*
	No	16(94.1)	310(90.4)	45(97.8)	
Smoking					
	Yes	4(23.5)	138(40.2)**	9(19.6)*	0.012***
	No	13(76.5)	205(59.8)*	37(80.4)**	

* Pearson's exact chi-square;

** Pearson's asymptotic chi-square;

*** Mann-Whitney test

A comparison was made between the histology and cytology variables for the age group recommended and those not recommended by the Brazilian Guidelines for Cervical Cancer Screening (Table 4). There was a greater relationship between invasive cancer histology and the over-64 age group, the proportion of CIN 2 was higher in the under-25 age group, and CIN 3 in the 25-64 age group, with statistical significance. As for the cytology of origin, the proportion of ASC-US and LSIL was higher in the group aged under 25 and the proportion of ASC favoring neoplasia and HSIL was higher in the group between 25 and 64 years old. The proportion of epidermoid carcinoma and invasive adenocarcinoma was higher in the group over 64, with statistical significance. In the other cytology, there was no difference at the 0.05 level. Of the three women under the age of 25 who presented histology for cancer, only one had cytology of known origin (HSIL), the others had no cytology and had already been diagnosed with a vegetating lesion. The histopathology reports of the biopsies revealed two cases of moderately differentiated squamous cell carcinoma and one of poorly differentiated squamous cell carcinoma. Two women had no record of their disease staging. The only woman who had a record already had advanced disease, with stage IIIB.

**Table 4:** Comparison of the variable's histology and cytology of origin between women aged < 25 years, between 25 and 64 years, and > 64 years

Variables		CIN2+ <25 y n=17 n(%)	CIN2+ 25-64y n=343 n(%)	CIN2+>64 n=46 n(%)	p-value
Histology					
	CIN2	5(29.4)**	40(11.7)	1(2.2)*	<0.001***
	CIN3	9(52.9)	189(55.1)**	12(26.1)8	
	CA	3(17.6)	114(33.2)*	33(71.7)**	
Source cytology					
	Normal	1(6.7)	5(1.7)	0(0.0)	<0.001****
	ASC-US	3(20.0)**	9(3.1)	0(0.0)	
	AGC	0(0.0)	2(0.7)	1(2.9)	
	ASC favoring neoplasia	0(0.0)	22(7.6)**	0(0.0)	
	LSIL	2(13.3)	13(4.5)	0(0.0)	
	HSIL	9(60.0)	192(66.7)**	13(38.2)*	
	Epidermoid CA	0(0.0)	38(13.2)*	17(50.0)**	
	Adenocarcinoma?	0(0.0)	6(2.1)	3(8.8)**	
	Adenosquamous CA	0(0.0)	1(0.3)	0(0.0)	

* Adjusted residual <=-1.96;

** Adjusted residual >= +1.96;

*** Pearson's exact chi-square;

**** Pearson's asymptotic chi-square

Among the 406 patients included in this study, all diagnosed with CIN 2+, 15.5% were either younger than 25 years old or older than 64 years. Demographic and epidemiological data revealed that the median age of sexual debut was 14 years old. Multiparity was more frequent in women over 64. The use of condoms and estrogen-containing contraceptives was more common among women under 25. Smoking was more common in the age group recommended for screening (between 25 and 64 years old). As for the cytology of origin, ASC-H and HSIL were more frequent in the group included in the screening recommended by the Brazilian Guidelines. Cytology suggestive of epidermoid carcinoma and adenocarcinoma was higher in the group of women aged over 64 and the group under 25 had a higher proportion of ASC-US and LSIL. A higher proportion (71.7%) of invasive cancer histology was observed in the group aged over 64, the proportion of CIN 2 was higher in the group aged under 25 (29.4%), and CIN 3 in the group aged between 25 and 64 (55.1%).

## Discussion

In this study, the proportion of HSIL in women younger than 25 years old was 2.21% (9), with exclusively HSIL cytology. In this age group, 3.45% (14) of histological results showed CIN 2 or 3. Available studies correlate cytology reports with histopathology findings of CIN 2+. Thus, cytology can be classified based on its level of suspicion as either low-grade or high-grade for squamous intraepithelial lesions of the cervix.

Prior cytology findings demonstrated a strong correlation with histopathological CIN 2+ diagnoses, with HSIL cytology being the most prevalent precursor across all histologic categories. Among women diagnosed with CIN 2, 63% had prior cytology classified as HSIL, while 17.4% had LSIL, reinforcing the role of cytology as an essential screening tool for high-grade lesions. In CIN 3 cases, HSIL was found in 79% of prior cytology reports, whereas 4.8% had findings consistent with squamous cell carcinoma, indicating that cytology alone may not always capture high-grade lesions at their earliest stages. Women diagnosed with invasive carcinoma showed a distinct cytological profile, with 30% presenting prior cytology suggestive of squamous cell carcinoma and 12.7% with HSIL, highlighting the progression from high-grade lesions to invasive disease. In younger women under 25 years, all CIN 2+ cases had prior HSIL cytology, reinforcing the transient nature of some lesions in this age group despite high-grade cytological findings. In contrast, among women older than 64 years, 8.37% had prior HSIL cytology and 3.2% had histopathological confirmation of high-grade lesions, emphasizing the risk of late diagnoses due to inadequate lifetime screening. Additionally, the presence of negative cytology prior to CIN 2+ diagnosis in 2.2% of CIN 2 cases and 2.7% of invasive carcinoma cases suggests limitations in the sensitivity of cytological screening and the potential benefit of incorporating HPV testing. The observed delay in histopathological confirmation and treatment, even in cases with high-grade cytology, may contribute to the persistence of cervical cancer cases, underscoring the need for more efficient follow-up protocols.

However, it is important to acknowledge that, as this is a cross-sectional study, it does not establish causality but rather provides epidemiological evidence supporting the transient nature of CIN 2 lesions in young women and the higher risk of progression to invasive carcinoma in older women. Our findings show that CIN 2 is more frequently diagnosed in women under 25 years old (29.4%), while CIN 3 and invasive cancer predominate in older age groups. This aligns with previous studies demonstrating that CIN 2 in young women often regresses spontaneously, whereas high-grade lesions in older women are more likely to persist and progress. Additionally, our data indicate that young women diagnosed with CIN 2+ had a higher proportion of prior high-grade cytology, whereas older women had a greater proportion of late-stage diagnoses, reinforcing the role of inadequate screening over a lifetime. While our study does not establish a causal association, these findings contribute to the broader body of evidence suggesting that extending screening to women under 25 years may lead to overtreatment of lesions that would otherwise regress naturally. Future longitudinal studies should further evaluate this progression to strengthen recommendations on screening policies.

In ASC-US cases, a Brazilian study found 1.85% of patients had CIN 2+, indicating a low correlation. In contrast, ASC-H showed a higher correlation, with 19.29% of cases presenting CIB 2+.^(3)^ For AGC reports, the correlation with CIN 2+ varies widely, ranging from 8.3 to 62.5%, depending on the type of AGC. This correlation is higher in high-grade AGC (AGC-H) and depends on the age group studied, being more frequently observed in postmenopausal women.^(4)^ In LSIL cases, the correlation with CIN 2+ remains low, typically below 2%.^(5)^ Conversely, in HSIL cases, the correlation is significantly higher, reaching up to 96.4%.^(6)^ For atypical cells of undetermined origin, since there is no definition of the cellular origin, it is assumed that it result from adenosquamous alterations, which are compatible with a high-grade lesion. However, no studies have specifically evaluated these results.^(2)^

In 2022, a systematic review and network meta-analysis evaluated the accuracy of various screening strategies for identifying cervical intraepithelial neoplasia grade ≥ 2 in healthy asymptomatic women. The findings suggest that combining different screening methods may improve accuracy by mitigating the limitations of standalone cytological or high-risk HPV tests, thereby reducing false-negative and false-positive rates.^(7)^

In Brazil, the average age of sexual debut is 14.9 years. However, in cases involving risk factors such as sexual violence, this first intercourse often occurs even earlier, generally around13 years of age. As a result, HPV infection and its prevalence tend to manifest earlier, since viral infection occurring within 2 to 3 years after the onset of sexual activities in 50 to 80% of adolescents.^(8–11)^

Moore et al.^(12)^ reviewed 511 medical records to assess the prevalence, progression, and regression rates of CIN 2 and 3 in adolescents. The authors concluded that the majority of girls with HSIL indeed had CIN 2 and the majority have experienced regression. They support continued vigilance in the assessment of adolescents but suggest that less intervention may be appropriate for CIN 2 cases.

In 2007, Fuchs et al.^(13)^ conducted a cohort study of 93 adolescents with CIN 2 to observe regression rates. Younger females (under 16 years of age) tended to have a shorter time to regression. Based on these findings, they concluded that due to the significant regression of CIN 2 cases in adolescent women, primary management in this population should consist of follow-up with cytology and colposcopy rather than immediate intervention.

In 2010, Moscicki et al.^(10)^ described the natural history of CIN 2 in adolescents and young women aged 13 and 24 years in a prospective study, with evaluation at 4-month intervals. The lesion regressed in 38% of cases within 1 year, in 63% within 2 years, and in 68% within 3 years. Factors associated with non-regression included the use of combined hormonal contraception and the persistence of any HPV type. Lesion progression at the end of the third year was observed in 15% of the young women. HPV 16/18 persistence and HPV 16/18 status at the last visit were associated with progression. The authors concluded that the high regression rate of CIN 2 supports the clinical observation of this type of lesion in adolescents and young women may not require immediate treatment but rather careful monitoring.^(14)^

The U.S. cancer prevalence studies have shown that adolescents rarely develop carcinoma. According to the most recent Surveillance, Epidemiology, and End Results Program USA statistics (2017-2022), the incidence of carcinoma in women younger than 20 years old is 0.1 per 100.000. The low rates of carcinoma suggest that, even in young women diagnosed with CIN 2 or 3, progression to cancer is rare. On the other hand, the incidence of carcinoma does not begin to rise until age 25, which supports more aggressive screening at that point. Cervical cancer is most frequently diagnosed among women aged 35-44.^(15)^

A compatible result was also obtained in a Campinas State University study in 2020, where the rate of women diagnosed with cervical cancer under the age of 25 was 1.5% of all registered cases. These younger patients had a shorter time since sexual debut, with more advanced disease staging and a higher proportion of non-squamous histology when compared to older women.^(14)^ Common situations in the Brazilian population could explain the relatively high number of cervical carcinoma cases in young women, which increases the risk of acquiring HPV, and a higher proportion of adolescents using estrogen hormonal contraceptives, which are believed to facilitate the oncogenesis of HPV 16. Aditionally, the progression from precancerous lesions and the development of cervical cancer in young women may be shorter than the classic period of 10 to 15 years, with an average time of 6.9 years between sexual debut and the diagnosis of cervical neoplasia.^(16)^

The proportion of invasive carcinoma in women under 25 years old was 0.74%, lower than the rates reported in the literature. The three patients with cancer histology had aggressive histological types and were therefore untraceable, which does not justify extending screening to younger patients.

Postmenopausal women with no previous diagnosis of cervical disease are also at low risk of developing cancer. Approximately 1 in 5 new cases of cervical cancer are diagnosed in women over the age of 65.^(17)^ Therefore, screening has been discontinued for women over the age of 64 who have had at least two consecutive negative tests in the last 5 years, or who have no history of cervical disease.^(2)^

Because cervical cancer is uncommon in adequately screened populations within this age group, most diagnoses are related to inadequate screening and/or lack of screening. The criteria for stopping screening appear to be highly reliable.^(18)^

The higher risk of cervical carcinoma in women over 60 is related to the difficulty in detecting early cell abnormalities that are common in the postmenopausal period.^(19)^ HPV testing would be of great importance since 70% of these women have a negative HPV test. There is also hypoestrogenism, which makes the squamocolumnar junction inaccessible to colposcopic visualization, making even cytology difficult. This factor, combined with benign changes, atrophy, hypotrophy, and inflammatory reactions, makes cervical cancer diagnosis more challenging in older women, even if they have regular gynecological check-ups.^(19)^ The use of topical estrogens for the proper collection and evaluation of screening cytology could be considered to reduce the dubious results related to hypoestrogenism.^(19)^

A study carried out in Vitória, Espírito Santo - Brazil, found a percentage of 70.9% of cases of cervical carcinoma in elderly women who were illiterate or had incomplete primary education. This finding highlights that this disease is directly related to advanced age and is associated with low socioeconomic status and limited education, which consequently results in the neglect of screening tests.^(20)^

In this study, the proportion of high-grade cytology in women older than 64 was 8.37%, with a predominance of invasive squamous cell carcinoma and HSIL. In terms of high-grade histologies, 3.2% were observed, while the proportion of invasive carcinoma was 8.12%. These data confirm that the finding of HSIL and cervical cancer in older women is a consequence of inadequate screening at the recommended age group.

The findings of this study are based on a sample of women attended at a tertiary care university hospital, which may introduce selection bias. Women treated in this setting are often referred for more complex cases or advanced disease stages, potentially leading to an overrepresentation of severe outcomes compared to those in primary care or rural populations. As a result, the generalizability of these findings to the broader population, including women with limited access to specialized care or those in different socioeconomic and geographic contexts, may be restricted. This limitation should be considered when interpreting the results, as they may not fully represent the epidemiological profile of cervical lesions in the general population. Further studies involving more diverse populations are recommended to validate these findings.

Despite the high rates of HPV infection and related cervical, the diagnosis of cervical cancer is uncommon and does not justify screening in women younger than 25 years old. Women over 64 years of age with two negative cytology tests in the last 5 years should also no longer be screened. The need to develop effective strategies for the prevention of cervical cancer in young women means that the cytopathologic examination and its monitoring are essential to determine the best time for subsequent interventions and benefits.

Young women who are sexually active should receive counseling on contraception, prevention of sexually transmitted infections, and safer sex practices. These measures can be implemented without the need for inclusion in the screening program. Since the relationship between persistent HPV infection, specially types 16 and 18, and the development of cervical cancer is already known, HPV DNA testing could increase the sensitivity of the method used for screening, especially in women aged 30 years and older, if available.

Organized cytologic screening, rather than opportunistic, is an important determinant of early detection and treatment of cervical cancer and its precursor lesions in order to achieve high coverage of the target population. It is the most important component in primary care to achieve a significant reduction in the incidence and mortality from cervical cancer, without burdening the health system with inadequate screening.

## Conclusion

Although this is a cross-sectional study and does not establish causality, it provides epidemiological evidence supporting the current screening guidelines. However, the findings also emphasize the necessity of long-term follow-up studies to further evaluate the natural history of CIN 2+ lesions in different age groups. Future research should explore the impact of alternative screening strategies, such as HPV-based testing, to optimize early detection and risk stratification while minimizing overtreatment in younger women and improving late-stage diagnosis prevention in older populations.
